# Association between serum soluble CD40 ligand levels and mortality in patients with severe sepsis

**DOI:** 10.1186/cc10104

**Published:** 2011-03-15

**Authors:** Leonardo Lorente, María M Martín, Nerea Varo, Juan María Borreguero-León, Jordi Solé-Violán, José Blanquer, Lorenzo Labarta, César Díaz, Alejandro Jiménez, Eduardo Pastor, Felipe Belmonte, Josune Orbe, José A Rodríguez, Eduardo Gómez-Melini, José M Ferrer-Agüero, José Ferreres, María C LLimiñana, José A Páramo

**Affiliations:** 1 Intensive Care Unit, Hospital Universitario de Canarias, Ofra, s/n, La Laguna - 38320, Santa Cruz de Tenerife, Spain; 2 Intensive Care Unit, Hospital Universitario Nuestra Señora de Candelaria, Crta del Rosario s/n, Santa Cruz de Tenerife - 38010, Spain; 3 Biochemistry Deparment, Clínica Universidad de Navarra, Avda Pío XII n°55, Pamplona - 31008, Spain; 4 Laboratory Deparment, Hospital Universitario de Canarias, Ofra, s/n, La Laguna - 38320, Santa Cruz de Tenerife, Spain; 5 Intensive Care Unit, Hospital Universitario Dr. Negrín, Barranco de la Ballena s/n, Las Palmas de Gran Canaria - 35010, Spain; 6 Intensive Care Unit, Hospital Clínico Universitario de Valencia, Avda. Blasco Ibáñez no. 17-19, Valencia - 46004, Spain; 7 Intensive Care Unit, Hospital San Jorge de Huesca, Avenida Martínez de Velasco n°36, Huesca - 22004, Spain; 8 Intensive Care Unit, Hospital Insular, Plaza Dr. Pasteur s/n, Las Palmas de Gran Canaria - 35016, Spain; 9 Mixed Research Unit HUC-ULL, Hospital Universitario de Canarias, Ofra, s/n, La Laguna - 38320, Santa Cruz de Tenerife, Spain; 10 Atherosclerosis Research Laboratory, CIMA-University of Navarra, Avda Pío XII no. 55, Pamplona - 31008, Spain; 11 Laboratory Department, Hospital San Jorge de Huesca, Avenida Martínez de Velasco no. 36, Huesca - 22004, Spain

## Abstract

**Introduction:**

CD40 Ligand (CD40L) and its soluble counterpart (sCD40L) are proteins that exhibit prothrombotic and proinflammatory properties on binding to their cell surface receptor CD40. The results of small clinical studies suggest that sCD40L levels could play a role in sepsis; however, there are no data on the association between sCD40L levels and mortality of septic patients. Thus, the aim of this study was to determine whether circulating sCD40L levels could be a marker of adverse outcome in a large cohort of patients with severe sepsis.

**Methods:**

This was a multicenter, observational and prospective study carried out in six Spanish intensive care units. Serum levels of sCD40L, tumour necrosis factor-alpha and interleukin-10, and plasma levels of tissue factor were measured in 186 patients with severe sepsis at the time of diagnosis. Serum sCD40L was also measured in 50 age- and sex-matched controls. Survival at 30 days was used as the endpoint.

**Results:**

Circulating sCD40L levels were significantly higher in septic patients than in controls (*P *= 0.01), and in non-survivors (*n *= 62) compared to survivors (*n *= 124) (*P *= 0.04). However, the levels of CD40L were not different regarding sepsis severity. Logistic regression analysis showed that sCD40L levels >3.5 ng/mL were associated with higher mortality at 30 days (odds ratio = 2.89; 95% confidence interval = 1.37 to 6.07; *P *= 0.005). The area under the curve of sCD40L levels >3.5 ng/mL as predictor of mortality at 30 days was 0.58 (95% CI = 0.51 to 0.65; *P *= 0.03).

**Conclusions:**

In conclusion, circulating sCD40L levels are increased in septic patients and are independently associated with mortality in these patients; thus, its modulation could represent an attractive therapeutic target.

## Introduction

Severe sepsis is a common, expensive, and frequently fatal condition, leading to as many deaths annually as acute myocardial infarction [1]. Thus, a continuous search for new biomarkers in sepsis is necessary to aid early diagnosis and stratification of its severity.

CD40 Ligand (CD40L) and its soluble counterpart (sCD40L) are proteins that exhibit prothrombotic and proinflammatory properties on binding to their cell surface receptor CD40 [2,3]. CD40L is a member of the tumour necrosis factor (TNF) family and is expressed as a transmembrane protein in activated platelets [4,5]. CD40L exerts several pro-inflammatory [6,7] and procoagulant [8-13] effects.

Higher levels of sCD40L have been found in patients with acute coronary syndrome [14,15], stroke [16], systemic lupus erythematosus [17], and chronic lymphocytic leukemia [18]. The role of sCD40L in sepsis has hardly been studied. In some animal models, an increase in sCD40L was reported after the development of sepsis [19,20]. In humans, higher sCD40L levels were found in 49 patients with meningococcal sepsis and 15 patients with African tick bite fever compared with controls [21,22]. In other small series with pulmonary tuberculosis, higher sCD40L levels were found in patients with more severe disease [23,24]. A study including 35 septic patients found higher circulating sCD40L levels in non-surviving than in surviving patients [25]; however, there are no data on the association between circulating sCD40L levels and mortality of septic patients.

We hypothesized that circulating sCD40L levels could be associated with an adverse outcome in patients with severe sepsis. The primary objective of this study was to determine the association between the circulating sCD40L levels and mortality, and the secondary objective to determine the association between circulating sCD40L, inflammatory and prothrombotic markers in these patients.

## Materials and methods

### Design and subjects

A multicenter, observational, prospective study was carried out in six Spanish ICUs. The study was approved by the Institutional Review Boards of the six hospitals and written informed consent from the patients or from the family members was obtained. A total of 186 patients with severe sepsis and 50 age- and sex-matched healthy controls were included.

The diagnosis of sepsis and severe sepsis was established according to the International Sepsis Definitions Conference [26]. Severe sepsis was defined as sepsis complicated by organ dysfunction. Sepsis was defined as a documented or suspected infection (defined as a pathologic process induced by a microorganism) and some of the following parameters: I) General parameters: fever (core temperature higher than 38.3°C), hypothermia (core temperature lower than 36.0°C), tachycardia (heart rate higher than 90 beats/minute), tachypnea (respiratory rate higher than 30 breaths/minute), altered mental status, significant edema or positive fluid balance (higher than 20 ml/kg over 24 hours), hyperglycemia (plasma glucose higher than 110 mg/dl) in the absence of diabetes; II) Inflammatory parameters: leukocytosis (white blood cell count higher than 12,000/mm^3^), leukopenia (white blood cell count lower than 4,000 mm^3^), normal white blood cell count with a percentage of immature forms higher than 10%, plasma C-reactive protein >2 standard deviations above the normal value, plasma procalcitonin >2 standard deviations above the normal value; III) Hemodynamic parameters: arterial hypotension (systolic blood pressure lower than 90 mmHg, mean arterial blood pressure lower than 70 mmHg, or decrease of systolic blood pressure from the baseline higher than 40 mmHg), mixed venous oxygen saturation higher than 70%, cardiac index higher than 3.5 l/min/m^2^; IV) Organ dysfunction: arterial hypoxemia (pressure of arterial oxygen/fraction inspired oxygen (PaO_2_/FIO_2_) ratio <300), acute oliguria (urine output <0.5 ml/kg/h for at least two hours), creatinine increase ≥0.5 mg/dl, thrombocytopenia (platelet count <100,000/mm^3^), hyperbilirubinemia (total bilirubin >4 mg/dl); V) Tissue perfusion parameters: hyperlactatemia (>3 mmol/l), decreased capillary refill or mottling.

Exclusion criteria were: age <18 years, pregnancy, lactation, human immunodeficiency virus (HIV), solid or haematological tumour, or immunosuppressive, steroid or radiation therapy.

### Variables recorded

The following variables were recorded for each patient: sex, age, diabetes mellitus, chronic obstructive pulmonary disease (COPD), site of infection, creatinine, leukocytes, lactatemia, platelets, Acute Physiology and Chronic Health Evaluation II (APACHE II) score [27], Sepsis-related Organ Failure Assessment (SOFA) score [28]. We assessed survival at 30 days as the endpoint.

Blood samples were collected from 186 patients with severe sepsis at the time of the diagnosis and from 50 age- and sex-matched controls.

### Serum levels of sCD40L

Venous blood samples were collected in serum separator tubes (SST) and centrifuged within 30 minutes at 1,000*g for 15 minutes. The serum was removed and frozen at -80°C until measurement. Serum sCD40L levels were assayed by specific ELISA (Bender MedSystems, Vienna, Austria) according to the manufacturer's instructions in the Atherosclerosis Research Laboratory of CIMA-University of Navarra (Pamplona, Spain).

### Plasma levels of TF

Venous blood samples were collected in citrate collected plasma tubes and centrifuged within 30 minutes at 1,000*g for 15 minutes. The plasma was removed and frozen at -80°C until measurement. Assays for TF antigen were performed by specific ELISA (Imubind Tissue Factor ELISA™, American Diagnostica, Inc, Stanford, CT, USA) in the Laboratory Department of the Hospital Universitario de Canarias (La Laguna, Santa Cruz de Tenerife, Spain).

### Serum levels of TNF-α and IL-10

Serum separator tubes (SST) were used to determine TNF-α and IL-10 serum levels. Venous blood samples were taken and centrifuged within 30 minutes at 1,000 *g *for 15 minutes, and the serum was removed and frozen at -80°C until measurement. TNF-α and IL-10 serum levels were measured by a solid-phase, chemiluminiscent immunometric assay kits (Immulite^®^, Siemens Healthcare Diagnostics Products, Llanberis, UK) in the Laboratory Deparment of the Hospital Universitario de Canarias (La Laguna, Santa Cruz de Tenerife, Spain).

### Statistical analysis

In a pilot study with 30 patients with severe sepsis, we found that surviving patients show lower circulating levels of sCD40L (3.83 ± 1.44 ng/mL) than non-survivors (4.37 ± 1.52 ng/mL). We calculated to include 186 patients in a cohort study to demonstrate significant differences in the circulating levels of sCD40L between groups, for a power of 80% and a 5% type I error rate.

Continuous variables are reported as medians and interquartile ranges. Categorical variables are reported as frequencies and percentages. Comparisons of continuous variables between groups were carried out using Wilcoxon-Mann-Whitney test. Comparisons between groups on categorical variables were carried out with chi-square test. The association between continuous variables was carried out using Spearman's rank correlation coefficient or Spearman's rho coefficient. The cut-off 3.5 ng/mL was selected using a likelihood method as previously described [29]. Receiver operation characteristic (ROC) curves using lactate, APACHE score, sCD40L ≥3.5 ng/mL as independent variables, and exitus at 30 days as a dependent variable were obtained. To calculate the standard error of the area under the curves we used the method of Delong *et al. *[30]. Survival curves at 30 days, using sCD40L levels ≥ or lower than 3.5 ng/mL, were represented using the Kaplan-Meier method and were compared by log-rank test. Multivariate logistic regression analysis was applied to determine the independent contribution of sCD40L levels, lactate levels, APACHE-II score and thrombocytopenia to the prediction of the mortality during the 30-day period. Odds ratio and its 95% confidence intervals were calculated as measurement of the clinical impact of the predictor variables. A *P*-value of less than 0.05 was considered statistically significant. Statistical analyses were performed with SPSS 17.0 (SPSS Inc., Chicago, IL, USA), NCSS 2000 (Kaysville, UT, USA), and Statistic 8.0 (Tulsa, OK, USA).

## Results

Baseline characteristics of 186 patients with severe sepsis and 50 age- and sex-matched controls are shown in Table 1. Higher sCD40L levels were observed in the group of patients with severe sepsis compared with controls (*P *= 0.01) (Table 1).

**Table 1:** Comparison between patients with severe sepsis and controls

	Controls (*n *= 50)	Patients with severe sepsis (*n *= 186)	*P*
Female sex -- n (%)	13 (26.0)	64 (33.3)	0.11
Age * (years)	57 (50 to 63)	60 (49 to 70)	0.39
sCD40L* (ng/ml)	3.29 (2.10 to 4.19)	3.97 (2.60 to 5.62)	0.01

* Median (25^th ^to 75^th ^percentiles).

Non-surviving septic patients (*n *= 62) showed higher sCD40L levels (*P *= 0.04) than survivors (*n *= 124) after the 30-day follow-up. Non-surviving patients also showed a higher incidence of diabetes mellitus (*P *= 0.02), higher lactatemia (*P *< 0.001), higher SOFA (*P *< 0.001) and APACHE-II (*P *< 0.001) scores, and lower platelet count (*P *= 0.002) and IL-10 (*P *< 0.001) than surviving patients (Table 2).

**Table 2:** Demographic and clinical parameters of survivors and non-survivors patients with severe sepsis

	Survivors (*n *= 124)	Nonsurvivors (*n *= 62)	*P*
Female sex *	39 (31.5)	25 (40.3)	0.25
Age (years) ^†^	55 (45 to 68)	63 (51 to 72)	0.19
Diabetes mellitus *	25 (20.2)	23 (37.1)	0.02
COPD *	19 (15.3)	9 (14.5)	0.99
Statins previous to sepsis diagnosis*	27 (21.8)	16 (25.8)	0.58
Ischemic heart disease*	14 (11.3)	7 (11.3)	0.99
Site of infection			0.90
Respiratory *	64 (51.6)	36 (58.0)	
Abdominal *	29 (23.4)	13 (21.0)	
Other sites*	31 (25.0)	13 (21.0)	
Source of sepsis			0.73
Community*	93 (75.0)	49 (79.0)	
Nosocomial extra-ICU*	12 (9.7)	7 (11.3)	
Nosocomial intra-ICU*	19 (15.3)	6 (9.7)	
Pa0_2_/FI0_2 _ratio ^†^	162 (107 to 260)	164 (96 to 228)	0.18
Creatinine (mg/dl) ^†^	1.2 (0.8 to 1.9)	1.4 (0.9 to 2.8)	0.06
Bilirubin (mg/dl) ^†^	0.9 (0.6 to 1.6)	0.9 (0.5 to 2.0)	0.64
Leukocytes/mm^3 †^	14200 (9200 to 18700)	15850 (9050 to 22525)	0.34
Lactatemia (mmol/L) ^†^	1.7 (1.0 to 3.5)	3.9 (1.3 to 6.7)	<0.001
Platelet count*10^3^/mm^3 ^^†^	199 (136 to 270)	139 (79 to 218)	0.002
APACHE-II score ^†^	19 (14 to 23)	23 (18 to 29)	<0.001
SOFA score ^†^	9 (7 to 11)	12 (9 to 14)	<0.001
Mechanical ventilation*	102 (82.3)	58 (93.5)	0.04
Septic shock*	105 (84.7)	57 (91.9)	0.24
sCD40L (ng/ml) ^†^	3.78 (2.51 to 5.38)	4.42 (3.05 to 6.09)	0.04
Tissue factor (pg/ml) ^†^	123 (99 to 163)	120 (96 to 150)	0.56
TNF-alpha (pg/ml) ^†^	32 (20 to 50)	39 (18 to 79)	0.38
IL-10 (pg/ml) ^†^	10 (6 to 36)	53 (7 to 169)	<0.001

* Variable expressed as frequency (%); ^†^variable expressed as median (25^th ^to 75^th ^percentiles).

APACHE, Acute Physiology and Chronic Health Evaluation; COPD, chronic obstructive pulmonary disease; IL, interleukin; Pa0_2_/FI0_2_, pressure of arterial oxygen/fraction inspired oxygen; SOFA, Sepsis-related Organ Failure Assessment score; TNF, tumour necrosis factor.

We did not find differences in 30-day survival between those patients that received statins before sepsis compared with those that did not receive statins (Table 2). After the diagnosis of sepsis, none of the patients continued receiving statins.

We did not find significant differences in sCD40L serum levels according to sex, diabetes mellitus status, COPD, use of statins before sepsis diagnosis, personal history of ischemic heart disease, need for mechanical ventilation and presence of septic shock (Table 3). Neither did we find significant differences in sCD40L serum levels according to the site and source of infection (Table 4).

**Table 3:** Serum levels of sCD40L according to clinical variables

	Yes patient number and sCD40L levels	Non patient number and sCD40L levels	*P*
Female	(*n *= 64) 4.39 (2.77 to 5.59)	(*n *= 122) 3.77 (2.51 to 6.02)	0.47
Diabetes Mellitus	(*n *= 48) 3.97 (2.81 to 5.38)	(*n *= 134) 4.08 (2.59 to 6.02)	0.85
COPD*	(*n *= 28) 4.35 (2.60 to 5.23)	(*n *= 154) 3.97 (2.65 to 6.02)	0.68
Statins previous to sepsis diagnosis	(*n *= 43) 3.84 (2.60 to 5.65)	(*n *= 139) 4.10 (2.69 to 5.70)	0.81
Ischemic heart disease	(*n *= 21) 4.10 (2.73 to 6.06)	(*n *= 162) 3.97 (2.62 to 5.60)	0.74
Mechanical ventilation	(*n *= 160) 4.75 (3.55 to 6.85)	(*n *= 26) 3.85 (2.59 to 5.59)	0.09
Septic shock	(*n *= 162) 4.54 (2.78 to 6.70)	(*n *= 24) 3.92 (2.60 to 5.60)	0.16
Survivors at 30 days	(*n *= 124) 3.78 (2.51 to 5.38)	(*n *= 62) 4.42 (3.05 to 6.09)	0.04

* COPD, chronic obstructive pulmonary disease.

**Table 4:** Serum levels of sCD40L according to the site and source of infection

	Patient number and sCD40L levels	*P*
Site of infection		0.52
Respiratory	(*n *= 100) 4.21 (2.81 to 6.09)	
Abdominal	(*n *= 42) 3.85 (2.59 to 5.36)	
Other sites	(*n *= 44) 3.83 (2.51 to 5.52)	
Source of sepsis		0.38
Community	(*n *= 142) 3.83 (2.55 to 5.46)	
Nosocomial extra-ICU	(*n *= 19) 4.70 (3.01 to 6.84)	
Nosocomial intra-ICU	(*n *= 25) 4.39 (2.91 to 6.18)	

Logistic regression analysis showed that serum sCD40L levels ≥3.5 ng/mL, lactatemia, APACHE-II score and platelet count <60,000/mm^3 ^were associated with death at Day 30 (Table 5).

**Table 5:** Multiple logistic regression analysis of variables to predict 30-day mortality

Variable	Odds Ratio	95% Confidence Interval	*P*
sCD40L levels >3.5 ng/mL	2.35	1.16 to 4.76	0.02
Lactatemia	1.19	1.06 to 1.34	0.004
APACHE-II	1.05	1.001 to 1.10	0.04

**Figure 1:**
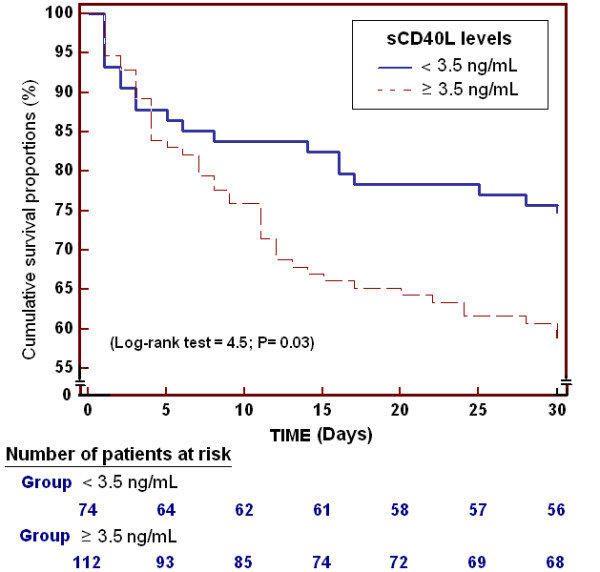
**Survival curves at 30 days using sCD40L levels higher or lower than 3.5 ng/mL**.

Survival analysis showed that patients with sCD40L levels ≥3.5 ng/mL presented higher mortality during the 30-day period than patients with lower levels (Chi-square: 4.50; *P *= 0.03) (Figure 1).

**Figure 2:**
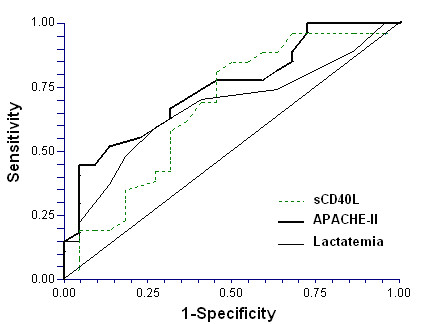
**Receiver operation characteristic analysis using sCD40L levels ≥ 3.5 ng/mL, APACHE-II and lactatemia as 30-days mortality predictors**.

The areas under the curves (AUC) for each predictor of mortality were the following: sCD40L ≥3.5 ng/mL (AUC = 0.58; 95% CI = 0.51 to 0.65; *P *= 0.03), lactatemia (AUC = 0.66; 95% CI = 0.59 to 0.73; *P *< 0.001) and APACHE-II (AUC = 0.70; 95% CI = 0.62 to 0.76; *P *< 0.001) (Figure 2).

**Figure 3:**
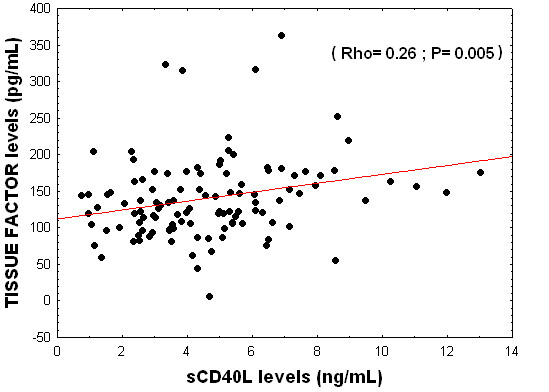
**Relationship between sCD40L and tissue factor levels**.

In the group of septic patients there were found an association between serum sCD40L levels and tissue factor levels (rho = 0.26; *P *= 0.005) (Figure 3) and platelet count (rho = 0.26; *P *< 0.001); but no association with lactatemia, TNF-α and IL-10 levels, SOFA or APACHE-II scores were observed (Table 6).

**Table 6:** Correlations between sCD40L levels (ng/ml) and lactatemia, APACHE-II, platelet count, TNF-alpha, interleukin-10 and tissue factor

	All patients (*n *= 186)	**Patients with platelet count ≥ 100,000/mm**^**3**^ (*n *= 138)	**Patients with platelet count < 100,000/mm**^**3**^ (*n *= 48)
Lactatemia (mmol/L)	Rho = -0.11 *P *= 0.15	Rho = -0.06 *P *= 0.50	Rho = -0.08 *P *= 0.61
APACHE-II (punctuation)	Rho = -0.03 *P *= 0.66	Rho = -0.07 *P *= 0.40	Rho = 0.20 *P *= 0.18
Platelet count (cells/mm^3^)	Rho = 0.25 *P *< 0.001	Rho = 0.22 *P *= 0.01	Rho = 0.09 *P *= 0.52
TNF-alpha^† ^(pg/ml)	Rho = -0.08 *P *= 0.31	Rho = 0.27 *P *= 0.76	Rho = -0.16 *P *= 0.28
Interleukin-10 (pg/ml)	Rho = -0.05 *P *= 0.49	Rho = 0.03 *P *= 0.70	Rho = -0.14 *P *= 0.35
Tissue factor (pg/ml)	Rho = 0.28 *P *= 0.002	Rho = 0.29 *P *= 0.006	Rho = 0.17 *P *= 0.42

*APACHE, Acute Physiology and Chronic Health Evaluation; ^†^TNF, tumour necrosis factor.

## Discussion

The main finding of the present study is that serum sCD40L levels were independently associated with mortality at 30 days in a large series of septic patients.

We found higher serum sCD40L levels in patients with severe sepsis than in healthy controls, in agreement with previous studies [21,22,25]. We also found higher circulating sCD40L levels in non-surviving than in surviving patients, as previously reported by Nolan *et al*. in a small series [25]. In addition, we found that serum sCD40L levels ≥3.50 ng/mL were associated with higher death during the 30-day period in the multiple logistic regression analysis. Impaired prognosis was previously reported in patients with acute coronary artery syndrome and higher sCD40L levels [31]; however, our study is the first reporting an impaired prognosis in patients with severe sepsis and higher sCD40L levels.

The role of sCD40L in sepsis remains unclear; but it is possible that there are similarities with findings observed in coronary artery disease [2]. CD40L is stored in α-granules in unstimulated platelets but rapidly translocates to the platelet surface when platelets are activated, where it is cleaved and released into circulation as sCD40L. The sCD40L binds to circulating monocytes through its receptor CD40, promoting their adhesion to vascular endothelium. The sCD40L also binds to CD40 on endothelial cell surfaces. Activated endothelial cells produce the overexpression of transcriptional factors such as nuclear factor-kappa B (NF-kß) [32], with subsequent up-regulation of proinflammatory and prothrombotic factors. Thus, sCD40L could have prothrombotic effects via induction of TF [8-11], diminishing thrombomodulin expression [10,11], and binding to the glycoprotein IIb/IIIa platelet receptor [12,13]. All these effects could facilitate the development of vascular thrombosis, organ dysfunction and death.

We report for first time an association between sCD40L and TF levels in patients with severe sepsis, which has been previously described in culture of vascular endothelial cells [8-11]. However, the observed association between sCD40L levels and mortality could not been explained by the TF levels, since there were no significant differences between non-surviving and surviving patients. It is possible that other reported prothrombotic effects of sCD40L, such as reduced thrombomodulin expression [10,11] and binding to the glycoprotein IIb/IIIa platelet receptor [12,13] could lead to vascular thrombosis and, finally, death in patients with severe sepsis.

We found a positive association between serum sCD40L levels and platelet count, possibly because platelets are the major source of sCD40L in circulation [4,5]. However, we did not find this association in thrombocytopenic patients, which may be explained by different underlying mechanisms in septic patients [33,34], such as immune destruction by platelet antibodies, hematophagocytosis in the bone marrow, reduced production due to bone marrow depression and non-immune destruction by the interaction of activated platelets with endothelium.

We failed to find an association between sCD40L levels and sepsis severity criteria such as lactatemia, APACHE II score, SOFA score, TNF-α or IL-10 levels. This is in agreement with the results by Nolan *et al. *[25] reporting no correlation between sCD40L and the concentrations of IL-6, IL-12 or APACHE II. The lack of correlation may be due to a true absence of relationship or that sCD40L levels are underestimated in more severe disease (for example, dilution, leakage to the intersticial space and urin, increased uptake at sites of inflammation, and so on). In addition, sCD40L would most likely response earlier to changes in inflammatory activity that the APACHE, which is a composite of multiple parameters.

Whereas the strength of our study was the relatively large sample size compared with previous reports assessing sCD40L levels in septic patients, some limitations should be recognized. We determined a single testing point for sCD40L levels and we were, therefore, unable to establish the time course of serum sCD40L levels. Data on other coagulation factors were not analyzed. We determined sCD40 levels only in serum and not in plasma samples to evaluate possible differences due to there has been reported higher sCD40L levels in serum than in plasma levels [35], and in platelet rich plasma than in platelet poor plasma [36,37]. There has been reporting the association between sCD40L levels and other cytokines as IL-12 or interferon-gamma; however, we have not explored this possible association [6,38]. Neither, we have explored procalcitonin to determine its association with sCD40L levels. There are been reported an association between sCD40L levels and severity of acute coronary artery syndrome [31] and between troponin and severity of sepsis [39-41]; however, we have not investigated markers of cardiac damage to explore its association with sCD40L levels. The time until the diagnosis of sepsis can influence the levels of sCD40L observed; however, we have not report it. The serum blood samples for the determination of sCD40L were obtained at moment of sepsis diagnosis and APACHE II was calculated at 24 hours of admission to ICU; thus, we did know if this time-gap can affect in the association between both variables. We have not found significant difference in the survival at 30 days with the use of statins previously to the diagnosis of severe sepsis; and it was not possible to explore the effect of this agent group during the sepsis because in all patients was it suspended.

From a therapeutic perspective, the use of sCD40L modulators could be used as a new class of drugs for the treatment of severe sepsis [42-46]. In one study including patients with coronary artery disease, the use of statins decreased circulating sCD40L levels [42]. Besides, the results of some studies suggest that the use of statins could improve the prognosis in patients with infectious episodes [43-46]. However, in some human and animal studies the use of antibody against CD40L was associated with platelet activation and thromboembolic complications [47-49].

## Conclusions

In conclusion, circulating sCD40L levels are increased in septic patients and are independently associated with mortality in these patients; thus, its modulation could represent an attractive therapeutic target.

## Key messages

• Patients with severe sepsis showed higher circulating sCD40L levels than healthy controls.

• Non-survivor septic patients showed higher circulating sCD40L levels than survivor ones.

• Modulation of circulating sCD40L levels could represent an attractive therapeutic target in sepsis.

## Abbreviations

APACHE: Acute Phisiology and Chronic Health Evaluation; ICU: Intensive Care Unit; sCD40L: soluble CD40 Ligand; SOFA: Sepsis-related Organ Failure Assessment score.

## Competing interests

The authors declare that they have no competing interests.

## Authors' contributions

LL was responsible of conceiving, designing and coordinating the study, making substantial contributions to acquisition of data, analysis and interpretation of data, and drafting the manuscript. MMM, JSV, JB, LL, CD, EP, FB, JMFA, JF and CL have made substantial contributions to acquisition of data and provided useful suggestions. NV, JO and JAR carried out the determination of sCD40L, and made substantial contributions to analysis and interpretation of data. JMB and EGM carried out the determination of tissue factor, TNF-alpha and IL-10, and made substantial contributions to analysis and interpretation of data. AJ made substantial contributions to analysis and interpretation of data. JAP contributed to study design, and made substantial contributions to analysis and interpretation of data. All authors read and approved the manuscript.
